# Experimental Study on Macro and Meso Characteristics of Steel-Slag-Based Cemented Backfill Due to Microbial Mineralization Action

**DOI:** 10.3390/ma17133165

**Published:** 2024-06-27

**Authors:** Fengwen Zhao, Jianhua Hu, Yinan Yang, Taoying Liu

**Affiliations:** 1 School of Resources and Safety Engineering, Central South University, Changsha 410083, China; dr_zfw94@163.com (F.Z.); 215511069@csu.edu.cn (Y.Y.); taoying@csu.edu.cn (T.L.); 2 Zijin School of Geology and Mining, Fuzhou University, Fuzhou 350108, China

**Keywords:** microorganism, backfill material, steel slag powder, macro and meso characteristics

## Abstract

Steel slag is an industrial solid waste, which can provide a new calcium source for microbial mineralization as it contains abundant calcium elements. This study treated cemented backfill material with microorganisms and steel slag to enhance its performance. The influence of microbial treatment on the strength, microstructure, and pore characteristics of the backfill was assessed using a strength test, nuclear magnetic resonance, scanning electron microscopy, and X-ray diffraction. The results indicate that (1) the microbial mineralization and the hydration reaction take place at the same time; (2) when the proportion of bacterial solution exceeded 50%, microorganisms excessively consumed Ca^2+^, which hindered the following hydration reaction; (3) the additional amount of bacterial solution added into the steel-slag-based cemented backfill material should be less than 50%, which increases the strength by up to 22.10%; (4) the excessive bacterial solution sharply reduces the strength of the backfill even by 21.41%; and (5) the addition of bacterial solution affects the pore characteristics. A 50% bacterial solution can make backfill reach its lowest porosity. The strength has an inversely proportional relationship with porosity, diameter, and roundness (σ = ax + b, a < 0).

## 1. Introduction

Steel slag (SS) is a by-product produced during the steelmaking process, and a large amount of SS is produced every year in China. The range of SS produced for each ton of steel produced is 0.15~0.20 t, which results in 150 million t of SS in China [1]. The accumulation of SS not only causes environmental pollution but also occupies a large amount of land [2,3]. Although there have been many studies on the reuse of SS, the utilization rate of SS in China is still very low, which causes serious environmental pollution problems and economic problems [4,5,6]. Increasing the utilization rate of SS is an important way to solve these problems. The main components of mine backfill materials are solid waste and cement [7], and its application amount in the mine is very large. Therefore, SS can be applied to the backfill. SS contains many cementitious substances, so it can replace high-cost cement. Although the cementitious properties of SS are weak, the mine does not have high requirements for the strength of backfill [6,8,9]. Therefore, SS can replace part of the cement to reduce the amount of cement used in the backfill [10,11], which can not only increase the utilization rate of SS but also solve the problem of high filling costs in mines. However, according to this method, the application amount of SS in the mine is not significant, and other ways are needed to increase the application amount of SS.

Microbial-induced calcium carbonate precipitation (MICP) technology is the technology of combining Ca^2+^ with CO_3_^2−^ under the action of microorganisms to form calcium carbonate precipitation. This technology is widely used in crack repair, such as concrete cracks, rock cracks, etc. [12,13,14]. Xiao Yao et al. used microorganisms to repair cracked rock masses, and the results showed that the rock cracks were repaired well, the permeability coefficient was reduced, and the mechanical characteristics were also improved [14]. There are some studies of the application of this technology in the field of backfill materials [15,16,17,18]. Xuejie Deng et al. used microorganisms instead of cement to prepare backfill samples, whose maximum strength could reach up to 17.95 MPa [18]. However, the calcium source they used was CaCl_2_, which requires an additional cost in engineering applications. Some scholars have also studied using other materials to provide calcium sources. Jingping Qiu et al. conducted a study using carbide slag and gypsum as calcium sources for microorganisms, and the result shows that adding an appropriate amount of microorganisms under appropriate conditions could still effectively improve the strength of backfill [19]. And, SS also contains rich calcium elements, which can provide a calcium source for microorganisms. This can not only solve the problem of microbial calcium sources but also increase the utilization rate of SS. In order to achieve the application of SS and microorganisms in backfill material, it is necessary to test the various properties of backfill. The properties that need to be tested for backfill materials usually include mechanical characteristics, pore characteristics, and microstructure. Among them, the mechanical characteristics were tested by uniaxial compression, and the pore characteristics and microstructure were tested by nuclear magnetic resonance (NMR), scanning electron microscope (SEM), and other techniques. Zhuen Ruan et al., using uniaxial compression, found that rice stalks could effectively inhibit the deterioration of the strength of cemented backfill caused by high sulfide [20]. Fengwen Zhao et al. analyzed the pore characteristics and microstructure of the backfill material using NMR and SEM techniques and found that lime can promote the hydration reaction of phosphogypsum, thereby affecting the pore characteristics [21].

Based on the foundation and shortcomings of the above studies, to explore the influence of SS as a microbial calcium source on the various properties of the backfill material, this study used tailings, SS, cement, deionized water, and bacterial solution as raw materials to prepare the backfill material, and uniaxial compression, NMR, SEM, and XRD techniques were used to test the various properties of the backfill sample, thereby the influence of microbial mineralization on the macro and meso characteristics of the backfill material was studied.

## 2. Materials and Method

### 2.1. Experimental Raw Materials

**Figure 1:**
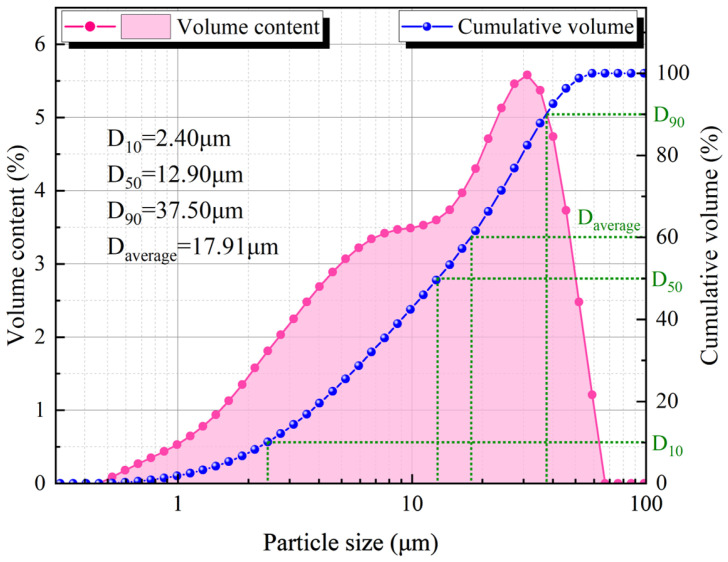
Particle size distribution of SS.

The tailings came from the Tiaoshuihe phosphate mine of Sanning Mining, the SS came from Guangxi Beibu Gulf Port Group, the ordinary Portland cement came from Changsha cement retail store, and the water was deionized water. The sieving method and laser particle size analyzer were used to analyze the particle size of the tailings and SS, and the results are shown in Table 1 and Figure 1. The chemical composition of SS was analyzed by X-ray fluorescence spectrometer (XRF) and X-ray diffraction (XRD), and the results are shown in Table 2.

### 2.2. Bacterial Solution

**Figure 2:**
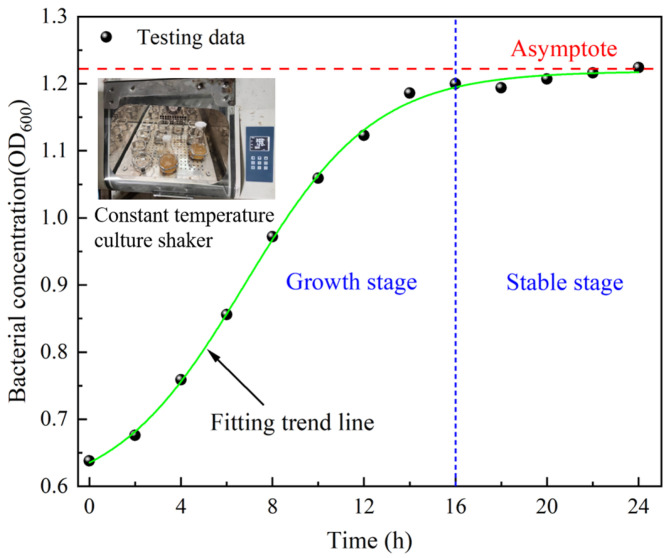
Microbial growth curve.

Studies have shown that Bacillus pasteurii is the most suitable microorganism for mine application [15,19,22]. Microorganisms need to be cultured and expanded before use. The culture process is as follows: the microorganisms are transferred to the culture medium and then placed in a constant temperature shaker for culture; finally, the cultured bacterial solution is added to the backfill material. During the culture process, the optical density (OD) value of the bacterial solution is measured every 2 h by a visible spectrophotometer, and the results are shown in Figure 2. The bacterial solution includes two parts: microorganisms and culture medium. The ratio of culture medium is tryptone/soy peptone/sodium chloride/urea/water = 3:1:1:4:200.

### 2.3. Experimental Scheme

**Figure 3:**
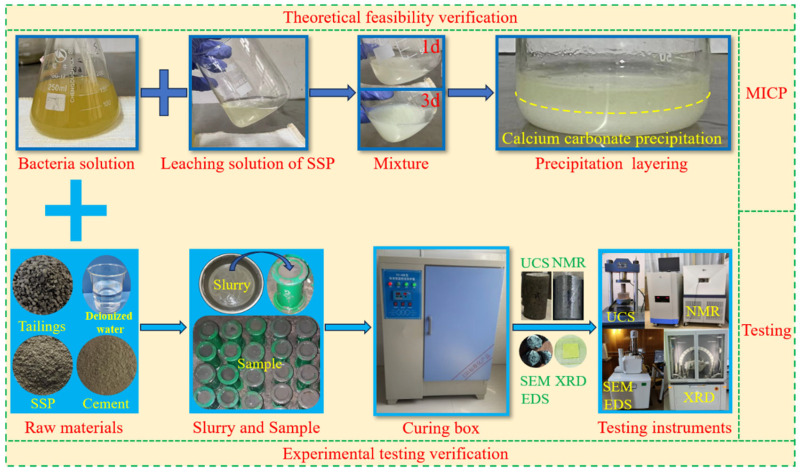
The experimental process.

In this experiment, tailings were used as aggregate, cement as cementitious material, and SS as a microbial calcium source. The preparation and curing processes of the backfill sample are as follows: First, the raw materials were mixed into slurry according to the ratio, and the slurry with different ratios was made into standard samples using φ50 mm × 100 mm cylindrical molds. The samples were labeled as A0–A4 according to the proportion of bacterial solution in the mixed solution—12 samples in each group; a total of 60 samples. The mixed solution includes bacterial solution and deionized water. The proportion of bacterial solution in the mixed solution is 0%, 25%, 50%, 75%, and 100%, respectively. The cement/sand ratio is 1:4 and the mass concentration is 80%; SS accounts for 28% of the total SS and tailings. The prepared samples were put into the curing box for curing, and all tests were carried out on the 3rd and 7th day of sample curing. The experimental process is shown in Figure 3.

### 2.4. Experimental Methods

Mechanical methods: According to the standard (GB/T 50081-2019) [23], the mechanical test is as follows. The samples that have been cured to a certain age are placed at the center of the pressure plate of the uniaxial compressor, and then the parameter (loading rate of 0.1 kN/s) is set for the mechanical test.

Methods of instrumental analysis: They include SEM, energy-dispersive X-ray spectroscopy (EDS), XRD, and NMR. For SEM, 1 cm^2^ fragments are selected at the center of the broken sample after uniaxial compression, then dried and sprayed gold, and finally put in the SEM analyzer for observation by chosen different magnification and EDS analysis. For XRD, the fragments are selected at the center of the broken sample after uniaxial compression, then ground and sieved, and finally formed into a powder to prepare a sample for testing. For NMR, the samples are processed by saturating them in water, then wrapped them with cling film, and finally put them into equipment for NMR testing after setting the parameters.

## 3. Result and Discuss

### 3.1. Mechanical Characteristics

**Figure 4:**
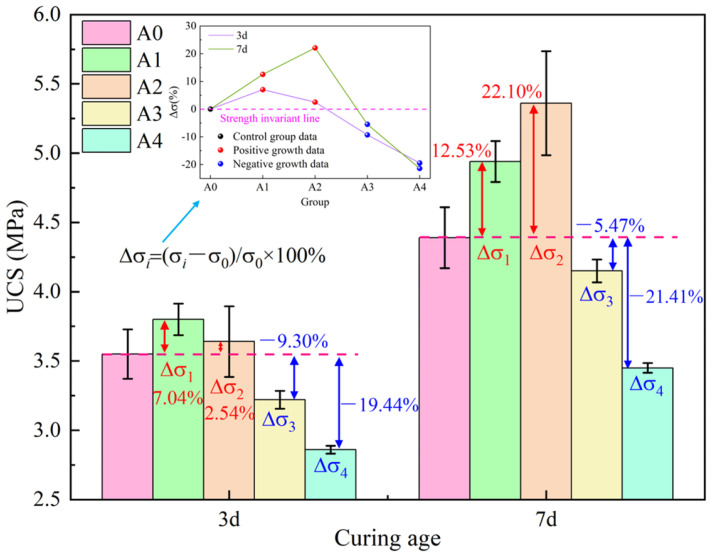
The strength characteristics of the backfill material.

Figure 4 shows the strength characteristics of the backfill material after adding different amounts of bacterial solution at different ages. It can be seen from Figure 4 that as the bacteria solution content increases, the strength of the backfill shows a trend of first increasing and then decreasing at 3 d and 7 d. The group A1 has the highest strength at 3 d and the group A2 has the highest strength at 7 d. This is because both MICP and the hydration reaction reach the maximum when the content of the bacterial solution is appropriate; the strength is maximal at this time. However, when the content of bacterial solution is too high, MICP consumes the Ca^2+^ required for the cement hydration reaction, resulting in a hindered cement hydration reaction—the hindered effect is greater than the filling effect, so the strength is reduced. When the strength reaches the maximum, the required bacterial solution content for 7 d is higher than that for 3 d. The reason is that the content of Ca^2+^ leached from SS increases with time [24], which provides more Ca^2+^ for the hydration reaction and microbial mineralization. To further analyze the effect of microorganisms on the strength of backfill materials, the strength growth rate is introduced for analysis, and its expression is as follows:

(1) $${∆\sigma }_{i}=\frac{\sigma_{i}-\sigma_{0}}{\sigma_{0}}\times 100\%$$

where Δ*σ_i_* represents the strength growth rate (%), *σ_i_* represents the strength of each group (MPa), and *σ*_0_ represents the strength of group A0 (control group) (MPa). The strength growth rate is calculated according to Formula (1), and the result is shown in Figure 4. The strength growth rates of groups A1 and A2 are both positive, indicating that the bacterial solution has a beneficial effect on strength. The strength growth rates of groups A3 and A4 are both negative, indicating that excessive addition of bacterial solution is harmful to strength.

### 3.2. Component Analysis

#### 3.2.1. XRD

**Figure 5:**
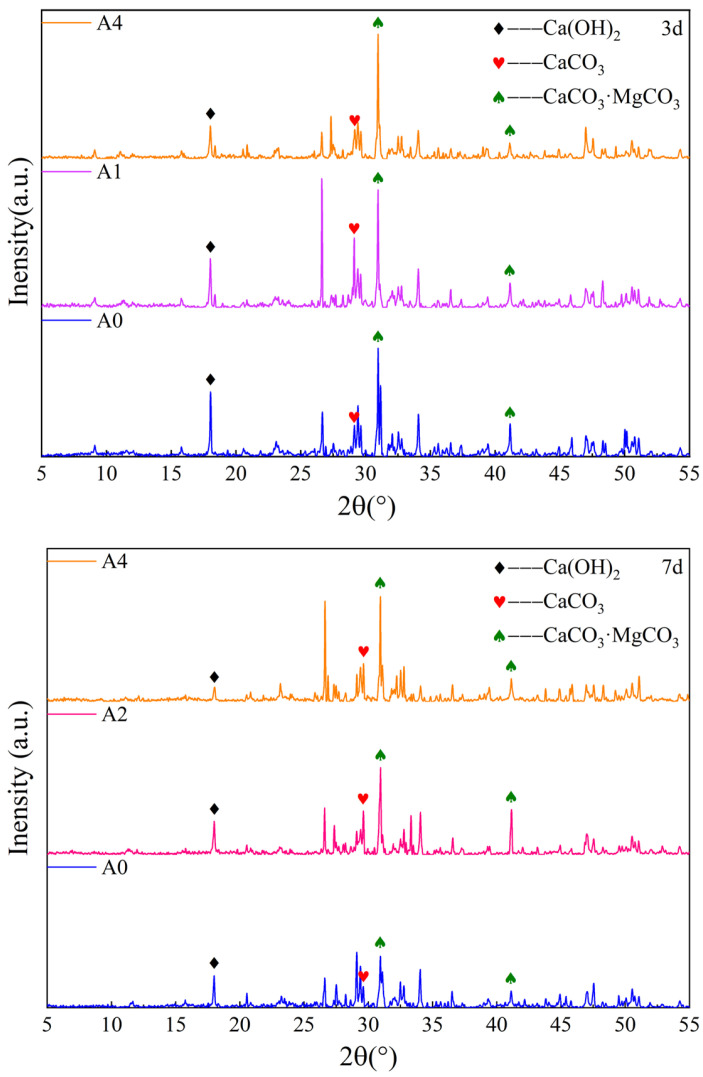
XRD characteristics.

XRD, as a commonly used method for substance composition testing, was used to test the substance composition of backfill materials with different bacterial solution content, and the results are shown in Figure 5. As can be seen from Figure 5, Ca(OH)_2_ content gradually decreases with the increase in bacteria solution content at 3 d, indicating that Ca^2+^ was consumed by microorganisms. However, the CaCO_3_ content first increases and then decreases, while the content of dolomite increases, indicating that when the CaCO_3_ content reaches a certain concentration, it will convert to dolomite. A similar law appeared at 7 d. According to the principle of MICP technology, the related reaction equations are as follows [16,22]:

(2) $$\;\;\;\;\;\;\;\;\;\;\;\;\;\;\;CO{\left(NH_{2}\right)}_{2}+2H_{2}O\overset{Bacterial}{\to }2NH_{4}^{+}+CO_{3}^{2-};$$

(3) $${Ca}^{2+}+{CO}_{3}^{2-}\overset{cell}{\to }Ca{CO}_{3}↓;$$

(4) $$\;\;\;\;\;\;\;\;\;\;\;\;\;\;\;\;\;\;Ca{CO}_{3}+{Mg}^{2+}+{CO}_{3}^{2-}\overset{cell}{\to }Ca{CO}_{3}\cdot Mg{CO}_{3}$$

where CO(NH_2_)_2_ represents urea and CaCO_3_·MgCO_3_ represents dolomite.

#### 3.2.2. SEM

**Figure 6:**
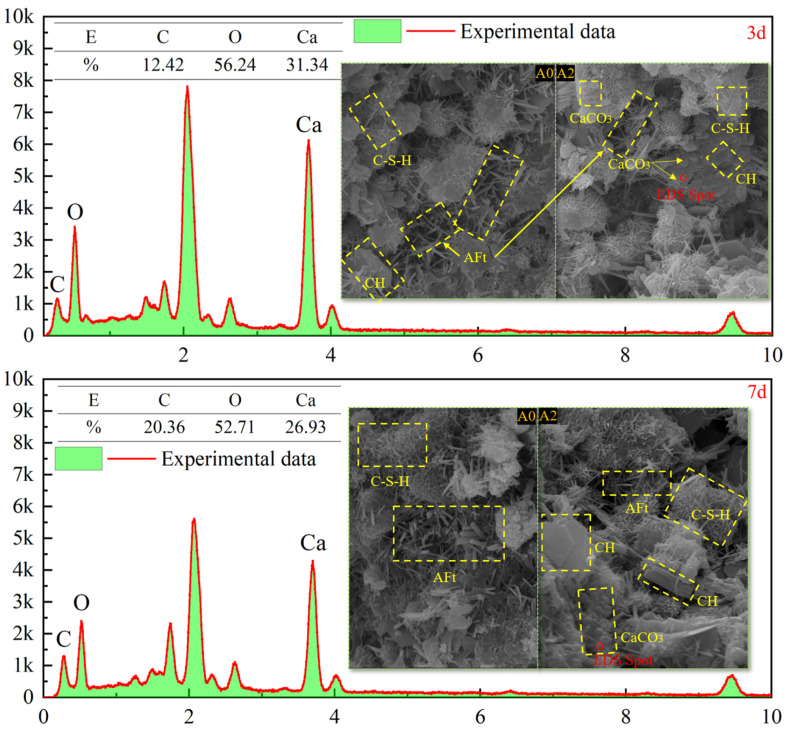
SEM and EDS images.

Figure 6 shows SEM and EDS images magnified 5000 times at 3 d and 7 d. Products with various morphologies can be clearly seen from the SEM images, and the structural composition can be determined according to the morphologies of the products. According to the research results of other scholars, it can be determined that the needle-shaped crystal is AFt, the gel state is C-S-H, and the layered structure is CH [25]. However, the crystal structure of CaCO_3_ is uncertain, and there are usually three crystal structures: aragonite, vaterite, and calcite, which have needle shaped, spherical, and diamond shaped morphologies, respectively [26]. It can be seen from Figure 6 that most products are AFt and C-S-H, because cement and SS contain a large number of C_2_S, C_3_S, and C_3_A. According to the hydration reaction equation [27,28], their hydration products are AFt and C-S-H. Due to different calcium sources, the morphology of CaCO_3_ is also different [29]. EDS technology was used to detect that the morphology of CaCO_3_ with SS as a calcium source is similar to that of vaterite. Comparative analysis of hydration products between different groups at different ages showed that group A0 had more AFt than group A2, but no CaCO_3_ was found in group A0. This is because Ca^2+^ in group A2 is consumed by microorganisms after adding bacterial solution, which hinders the formation of Aft but produces CaCO_3_.

### 3.3. Pore Characteristics

#### 3.3.1. Pore Characteristics of NMR

**Figure 7:**
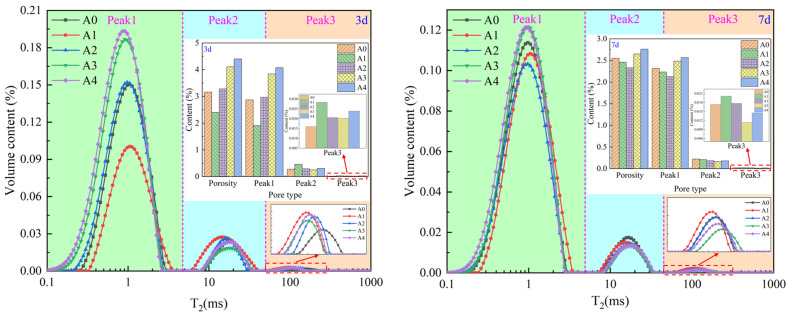
The pore distribution characteristics of the backfill material.

Figure 7 shows the pore distribution characteristics of the backfill material at 3 d and 7 d. It can be seen from Figure 7 that the NMR spectra at both ages contain three peaks, indicating that there are three types of pores in the backfill material. According to the relationship between pore size and T_2_ [28,30], the three peaks can be divided into three categories based on pore size—large, medium, and small—with peak 1 being a small pore, peak 2 being a medium pore, and peak 3 being a large pore. In terms of content, the content of small pores is the greatest, and the content of large pores is the least. As the bacterial solution content increases, porosity and small pore content first decrease and then increase. This is because when the bacterial solution content reaches a certain amount, both microbial mineralization and hydration reactions are optimum, resulting in the best filling effect of the product and the smallest porosity. However, as the bacterial solution content increases, microbial mineralization continuously consumes Ca^2+^, leading to weakened hydration reactions. Moreover, the filling effect of mineralization products is not as good as that of hydration products, resulting in an increase in porosity.

#### 3.3.2. Pore Characteristics of SEM Image

**Figure 8:**
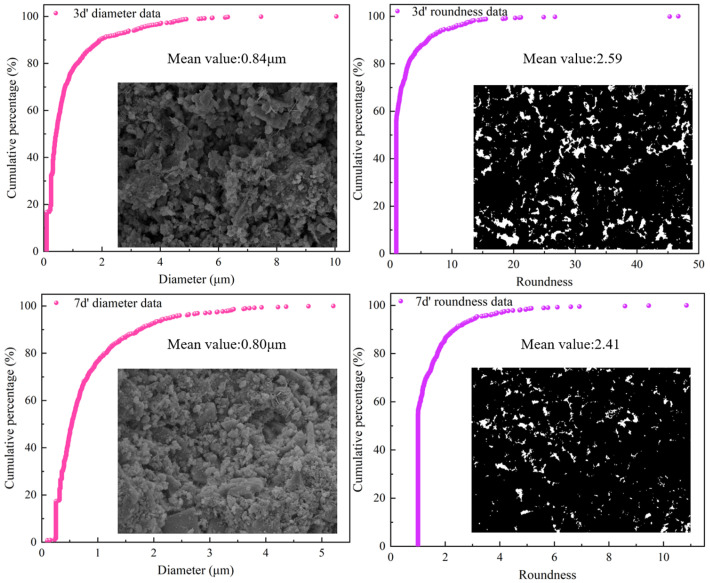
The pore parameter characteristic.

SEM can observe not only the microstructure but also the pore characteristics. When the magnification is low, pore distribution characteristics can be observed. Since it is difficult to distinguish between pores and solids by direct observation of SEM images, binarization processing is needed, and then digital image processing technology is used to obtain the parameter characteristics of pores. The results are shown in Figure 8 and Table 3. It can be seen from Figure 8 that the pore size of 70% of the pores is smaller than the average value, indicating that the pore size of most of the pores is small, which corresponds to the results of NMR. The roundness value of the pores is greater than 1.65, indicating that most pores are irregular. It can be seen from Table 3 that the pore parameter values at 7 d are all smaller than those at 3 d. This is because as the curing age increases, hydration reactions and microbial mineralization continue to occur, and the products fill the pores, causing them to become smaller and relatively regular.

### 3.4. The Influence of Pore Characteristics on Strength

#### 3.4.1. The Relationship between Porosity and Strength

**Figure 9:**
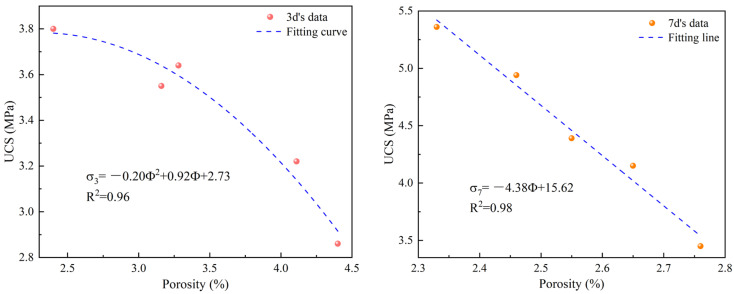
The relationship between porosity and strength.

MICP has a significant influence on the porosity of the backfill material, and there is a certain relationship between porosity and strength. Therefore, the relationship between the porosity and strength of backfill materials with different bacterial solution contents was established, and the results are shown in Figure 9. It can be found from the figure that there is a good relationship between them (R^2^ > 0.96), and they conform to the inverse proportional relationship, which can be expressed as follows:

(5) $$\sigma_{3}=-0.20\Phi^{2}+0.92\Phi +2.73;$$

(6) $$\sigma_{7}=-4.38\Phi +15.62$$

where σ_3_ and σ_7_ represent the strength at 3 d and 7 d, respectively (MPa); Φ represents porosity (%). From Equations (5) and (6), it can be found that they conform to a quadratic decreasing function at 3 d and a linear decreasing function at 7 d. MICP causes CaCO_3_ to fill the pores, resulting in a decrease in porosity and, thus, an increase in strength.

#### 3.4.2. The Relationship between Pore Shape Parameters and Strength

**Figure 10:**
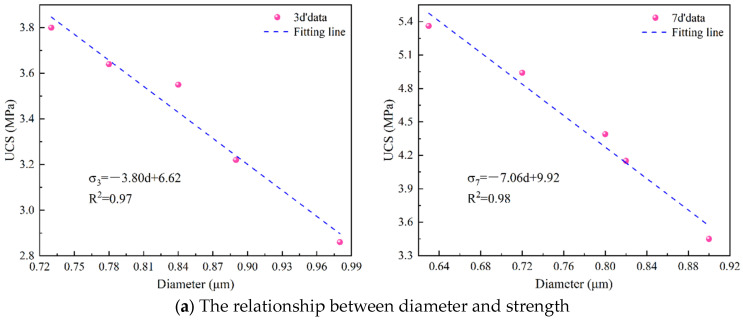

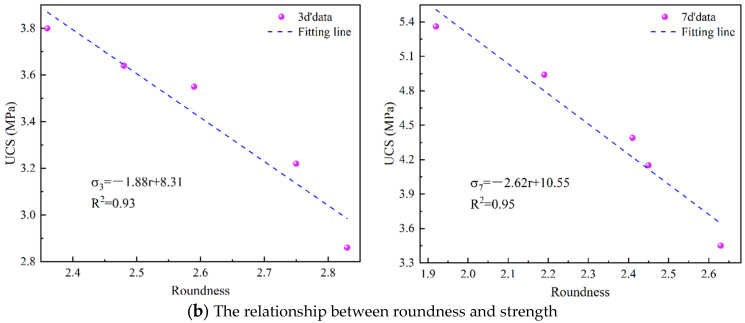
The relationship between pore shape parameters and strength.

The pore characteristics that affect strength include not only pore content but also pore shape. Pore size and pore shape are also the main factors affecting the strength. The relationship between pore shape parameters and strength was established, as shown in Figure 10. As can be seen from Figure 10, there is a good linear relationship between them (R^2^ > 0.93). Their relationship can be expressed as follows:

(7) $$\begin{array}{c} \sigma_{3}=-3.80d+6.62\\ \sigma_{7}=-7.06d+9.92 \end{array};$$

(8) $$\begin{array}{c} \sigma_{3}=-1.88r+8.31\\ \sigma_{7}=-2.62r+10.55 \end{array}$$

where *d* represents diameter (μm) and *r* represents roundness. It can be seen from Formulas (7) and (8) that the strength decreases with the increase in pore shape parameters. This is because the larger the pore size, the worse the support effect of the solid phase, and, thus, the lower the strength. Roundness represents the regularity of the pores, and the smaller the roundness, the more regular the pores. The more regular the pores, the higher the strength. Therefore, there is an inverse relationship between strength and roundness, which is consistent with the experimental results.

## 4. Conclusions

SS contains rich calcium elements, which can provide a new calcium source for microbial mineralization and, thus, also increase the utilization rate of SS. This study used SS as a calcium source to prepare backfill materials and then tested the various properties of the backfill samples using technologies such as uniaxial compression, NMR, SEM, and XRD, thereby studying the influence of microbial mineralization on the macro and meso characteristics of the backfill materials. The main conclusions are as follows:

Adding an appropriate amount of bacterial solution can improve the strength of the backfill. The strength of the backfill can be increased by up to 22.10% by adding an appropriate amount of bacterial solution to the steel-slag-base cemented backfill material. However, the excessive addition of bacterial solution can dramatically reduce the strength of the backfill, even by 21.41%.

The bacterial solution has an influence on the hydration reaction. The microbial mineralization and the hydration reaction were carried out at the same time. When the proportion of bacterial solution exceeded 50%, Ca^2+^ would be consumed excessively by microorganisms, which hindered the hydration reaction. As the curing age increases, there is continuous leaching of Ca^2+^, and the reaction will also continue.

There is an inversely proportional relationship between strength and pore characteristic parameters. The addition of bacterial solution affects the pore characteristics, thereby affecting the strength. A relationship model between strength and pore characteristic parameters is established, and the results show that there is an inversely proportional function relationship between strength and pore characteristic parameters, which can be expressed as σ = ax + b, a < 0.

## Figures and Tables

**Table 1:** Particle size distribution of tailings.

Particle Size/μm	<74	74–150	150–300	300–600	600–1180	1180–2360	2360–4750
Volume content/%	3.10	2.29	6.75	14.46	21.64	29.21	22.55
Cumulative volume/%	3.10	5.39	12.14	26.60	48.24	77.45	100

**Table 2:** Chemical composition of SS.

Element	Ca	O	Si	Mg	Al	Fe	Other
Content/%	37.46	36.70	14.88	1.91	0.81	0.53	7.71

**Table 3:** Mean values of pore parameters.

	Group	A0	A1	A2	A3	A4
3 d	Diameter/μm	0.84	0.73	0.78	0.89	0.98
	Roundness	2.59	2.36	2.48	2.75	2.83
7 d	Diameter/μm	0.80	0.72	0.63	0.82	0.90
	Roundness	2.41	2.19	1.92	2.45	2.63

## Data Availability

The data presented in this study are available on request from the corresponding author.
